# Identifying Subcellular Structure Components in *Escherichia Coli* by Crosslinking and SEC‐MS

**DOI:** 10.1002/pmic.70105

**Published:** 2026-01-21

**Authors:** Rachel A. Victor, Austin Lipinski, Paul R. Langlais, Jacob C. Schwartz

**Affiliations:** ^1^ Department of Pharmacology University of Arizona College of Medicine Tucson Arizona USA; ^2^ Department of Medicine Division of Endocrinology University of Arizona College of Medicine Tucson Arizona USA; ^3^ University of Arizona Cancer Center University of Arizona Tucson Arizona USA

## Abstract

Cells are comprised of a broad spectrum of structures that compartmentalize biochemical and signaling mechanisms. These structures can be comprised of many biomolecules, but especially lipids, proteins, and nucleic acids. Techniques are limited to quantify or discover new subcellular structures. We explored whether a proteomics approach using chemical crosslinking followed by size‐exclusion chromatography and mass spectrometry (SEC‐MS) of whole cell lysates can address this challenge. Formaldehyde crosslinking was used to preserve the weak molecular interactions responsible for many protein and nucleic acid assemblies. In this study, we perform the first formaldehyde crosslinking‐assisted SEC‐MS in a bacterial system. We demonstrate that when expressed ectopically in *E. coli*, large structures of a known assembly protein, FUS, can be detected through SEC‐MS. We then show that *E. coli* proteins are enriched in particles of large or medium size due to formaldehyde crosslinking, which is the first analysis by formaldehyde and SEC‐MS for a bacterial system. Last, analysis identified previously characterized *E. coli* protein assemblies and condensates, as well as potentially novel associations of prokaryote metabolism with large subcellular bodies. We propose this unbiased method can be used to stimulate or supplement targeted methods for discovery of new cellular bodies in a wide range of cell types.

## 1 Introduction

Some proteins can assemble in cells into large dynamic and multifaceted networks that underpin essential cellular functions, such as signal transduction, enzymatic reactions, and structural support [1, 2, 3]. Recent advances in structural biology and proteomics have greatly increased knowledge of how assemblies are formed, providing new insights into their roles in health and disease [4, 5, 6]. To date, most cell bodies were discovered serendipitously when a molecule of interest was observed by microscopy to be concentrated into puncta or another organized structure [7, 8]. The resolution limit for ordinary confocal microscopy is many times greater than a protein, allowing structures that have significance to molecular mechanisms and function to remain unnoticed [9, 10]. Advances in super‐resolution microscopy and live‐cell imaging offer powerful new tools to discover subcellular structures, however such methods remain largely a targeted approach to probe one or several molecules at a time across eukaryotes and prokaryotes [8, 11, 12, 13].

Study and discovery of subcellular structures by methods other than microscopy is challenging [8, 14]. The weak forces that make these structures dynamic do not allow them to survive cell lysis. Carefully optimized protocols allow some structures to be isolated nearly intact, such as stress granules [15], p‐bodies [16], nuclear pores [17], and the nucleolus [18]. Chemical crosslinking is a method to stabilize protein:protein and protein:nucleic acid interactions for study outside the cell [19, 20]. In addition to providing stability, covalent bonds can protect specific molecular interactions formed in the cell while strong detergents or sonication are used to dissociate aggregates formed in a cell lysate [21]. We have previously used size exclusion chromatography (SEC) and formaldehyde crosslinking to characterize subcellular particles comprised of heteronuclear RNA particle (hnRNP) proteins, such as FUSed in sarcoma (FUS), and RNA Pol II [14, 22, 23].

We reasoned that with the help of crosslinking, SEC combined with mass spectrometry analysis (SEC‐MS) could fill the gap to quantify and identify components of large subcellular structures or organelles. Current MS protocols have allowed quantitative analysis of whole cell or tissue interactomes [24, 25, 26]. SEC‐MS is an important method in the fields of proteomics, structural biology, and biopharmaceutical development [27, 28]. A strong precedence in published interactome studies demonstrates the compatibility of crosslinking with SEC‐MS [29], including formaldehyde crosslinking and label‐free SEC‐MS analysis [30], native complexes with SILAC‐based analysis (PCP‐SILAC) [31], cofractionation MS with and without crosslinking [32, 33], and in vivo crosslinking with cofractionation (X‐Co‐Frac‐MS) [34]. The key distinction of these previous studies is that they defined discrete complexes using SEC columns such as sec‐1000 or sec‐4000, which have the resolution to separate complexes somewhat larger than a ribosome [32, 33, 34]. We sought a census and relative abundance of proteins in loosely interacting and heterogeneous complexes or membrane‐less organelles, which require crosslinking to exist stably outside the cell, and we use a 10 × 300 mm CL2B column, which separates particles up to 400 nm or 10‐times the diameter of a single ribosome. Thus, for this study, we define discrete complexes of sizes up to that of a ribosome or spliceosome to be complexes of, “ordinary size”, or simply, “small” particles, and our focus is larger particles defined as, “medium” and, “large” [14, 23].

## 2 Materials and Methods

### 2.1 Cell Growth and Induced Expression

All FUS constructs were expressed with N‐terminally fused 6x His and maltose binding protein (MBP) to improve purification and solubility [35]. Growth of BL21(DE3) *E. coli* was performed essentially as previously described [36, 37]. Cells were transformed with MBP‐FUS or an empty vector plasmid, grown at 37°C with shaking until OD_600_ = 0.6, then cooled to 17°C before addition of IPTG (1 mM final concentration; GoldBio, cat. no. I2481C500) and allowed to grow overnight (16 h).

Cells recovered by centrifugation (4000 × g at 4°C) were washed with PBS, divided in half, then incubated 20 min at room temperature in PBS (uncrosslinked) or PBS with 1% (v/v) formaldehyde (crosslinked). Formaldehyde was quenched with (125 mM) glycine and cell pellets could be stored at −80°C. To prepare lysates, cell pellets were washed once with PBS and resuspended in *Escherichia coli* lysis buffer (1 M urea, 1 M KCL, 50 mM Tris‐HCl, pH 8, 1X protease inhibitors, 1.5 mM MgCl_2_) including 100 U Benzonase (Millipore Sigma, cat. no. E1014). Cells were sonicated using Bioruptor Pico (30 cycles on/off at 4°C, 30 s each; Diagenode, cat. no. B01060010). Lysates were cleared by centrifugation (>18,000 × g) for 30 min at 4°C. Apparent protein concentration was estimated by UV absorption at 280 nm.

### 2.2 Size Exclusion Chromatography

SEC analysis protocols used here have been previously published in detail [14, 23]. A total of 0.75 mg protein estimated by UV absorption was filtered by 0.45 µm PVDF centrifugal filter and injected into a Sepharose CL2B (Cytiva, cat. no. 17014001) 10 × 300 chromatography column equilibrated with 2 column volumes of SEC running buffer (20 mM HEPES, pH 7.9, 100 mM NaCl, 0.2 mM EDTA, 5% glycerol, 6 M urea, 0.5 mM DTT). Eluates were collected in 0.5 mL fractions and stored at −80°C. Particle size was measured by dynamic light scattering (Wyatt DynaPro Nanostar). Fractions were pooled according to size as large, medium, and small as described below.

For silver stain analysis of protein, SEC fraction pools were heated in 50 mM Tris‐HCl pH 7.4, 250 mM NaCl). Afterward protein was TCA precipitated by incubating 4 parts sample to 1 part TCA for 10 min on ice followed by 16,000 × g for 5 min at 4°C. Precipitate was washed twice with 1 mL acetone, dried at 95°C for 10 min, resuspended by 5 min sonication in 100 µL of 50 mM NH_4_HCO_3_ with 5 mM DTT then heated at 70°C for 30 min. SDS‐PAGE was performed with 4%–20% acrylamide gels and NuPAGE LDS Sample Buffer (Thermo Fisher Scientific, catalog no. NP0008). Silver staining was performed using the Pierce Silver Stain Kit (Thermo Fisher Scientific, catalog no. 24612) according to manufacturer's instructions.

### 2.3 ELISA

Protein in SEC fractions was immobilized in 96 well plates (Greiner Bio‐One, catalog no. 655074) by overnight incubation at 4°C. Wells were washed with TBS‐T (Tris‐buffered saline with 0.1% v/v Tween) and blocked with 5% nonfat dry milk in TBS‐T. Antibodies used were anti‐FUS (4H11, Santa Cruz, catalog no. sc‐47711) and goat antimouse IgG HRP (Fisher, catalog no. PI31432). Signal reading was with chemiluminescent substrate (Thermo Fisher Scientific, catalog no. PI37074) and by a BioTek Neo2 plate reader.

### 2.4 In‐solution Tryptic Digestion

Samples were TCA precipitated as described above, cooled to room temperature for 10 min, and incubated with 15 mM acrylamide for 30 min at room temperature while protected from light. The reaction was quenched with 5 mM DTT and incubated in the dark for 15 min. One µg of Lys‐C was added to each sample and incubated at 37°C for 2–3 h while shaking at 300 rpm followed by addition of 50 µL of 50 mM ammonium bicarbonate and 2 µg of trypsin and incubation overnight at 37°C while shaking at 300 rpm. 14.7 µL of 40% FA/1% HFBA was added to each sample and incubated for 10 min (final concentration is 4% FA/0.1% HFBA) to stop trypsin digestion. The samples were desalted with Pierce Peptide Desalting Spin Columns per the manufacturer's protocol (ThermoFisher Scientific, cat no. 89852) and the peptides were dried by vacuum centrifugation. The dried peptides were resuspended in 20 µL of 0.1% FA (v/v) and the peptide concentration was determined with the Pierce Quantitative Colorimetric Peptide Assay Kit per the manufacturer's protocol (ThermoFisher Scientific, cat no. 23275). 350 ng of the final sample was analyzed by mass spectrometry.

### 2.5 Mass Spectrometry and Data Processing

HPLC‐ESI‐MS/MS was performed as previously described in positive ion mode on a Thermo Scientific Orbitrap Fusion Lumos tribrid mass spectrometer fitted with an EASY‐Spray Source (Thermo Scientific, San Jose, CA) [38]. NanoLC was performed using a Thermo Scientific UltiMate 3000 RSLCnano System with an EASY Spray C18 LC column (Thermo Scientific, 50 cm × 75 µm inner diameter, packed with PepMap RSLC C18 material, 2 µm, cat. # ES803); loading phase for 15 min at 0.300 µL/min; mobile phase, linear gradient of 1%–34% Buffer B in 119 min at 0.220 µL /min, followed by a step to 95% Buffer B over 4 min at 0.220 µL /min, hold 5 min at 0.250 µL/min, and then a step to 1% Buffer B over 5 min at 0.250 µL /min and a final hold for 10 min (total run 159 min); Buffer A = 0.1% FA/H_2_O; Buffer B = 0.1% FA in 80% ACN. All solvents were liquid chromatography mass spectrometry grade. Spectra were acquired using XCalibur, version 2.3 (ThermoFisher Scientific).

### 2.6 Label‐free Quantitative Proteomics

Progenesis QI for proteomics software (version 2.4, Nonlinear Dynamics Ltd., Newcastle upon Tyne, UK) was used to perform ion‐intensity based label‐free quantification as previously described [39]. In brief, in an automated format, .raw files were imported and converted into two‐dimensional maps (*y*‐axis = time, *x*‐axis = *m*/*z*) followed by selection of a reference run for alignment purposes. An aggregate data set containing all peak information from all samples was created from the aligned runs, which was then further narrowed down by selecting only +2, +3, and +4 charged ions for further analysis. The samples were then grouped in crosslinked versus noncrosslinked. Peak lists of fragment ion spectra were exported in Mascot generic file (.mgf) format and searched against the Swissprot *E. coli* database (23149 entries) using Mascot (Matrix Science, London, UK; version 2.6). The search variables that were used were: 10 ppm mass tolerance for precursor ion masses and 0.5 Da product ion masses; digestion with trypsin; a maximum of two missed tryptic cleavages; variable modifications of oxidation of methionine and phosphorylation of serine, threonine, and tyrosine; ^13^C = 1. The resulting Mascot .xml file was then imported into Progenesis, allowing for peptide/protein assignment, while peptides with a Mascot Ion Score of < 25 were not considered for further analysis. Precursor ion‐abundance values for peptide ions were normalized to all proteins. For quantification, proteins must have possessed at least one or more unique, identifying peptide.

All proteins measured were exported from Progenesis. To calculate enrichment in a sample, the average XL signal was divided by the average No XL signal. Proteins were classified as enriched when log_2_(enrichment) was > 1. Gene ontology analysis was performed using Panther statistical overrepresentation tests compared to reference lists for all *E. coli* genes and significance determined by Fisher's exact test corrected for false discovery rate.

## 3 Results

### 3.1 Size Exclusion Chromatography of *E. coli* Cell Lysates

The human RNA‐binding protein FUS is well‐characterized to have assembly properties that can be studied in and out of the cell [40, 41]. Self‐assembly of human FUS into granules or aggregates has been studied in model organisms, including yeast, drosophila, and zebrafish [41]. We chose to express human FUS protein in *E. coli* as a model for a subcellular molecular assembly or nonmembrane bound organelle. This model offered the chance to confirm whether characterization by SEC‐MS matched previously published work in eukaryotic cells [23].

**FIGURE 1:**
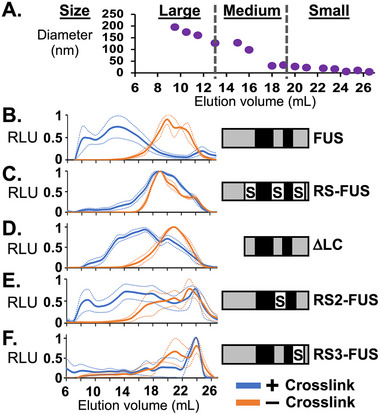
**FUS protein expressed in *E. coli* assembles into large particles. (A)** DLS measurement of particle sizes in cell lysate separated by SEC using a CL‐2b column. FUS protein in SEC fractions was measured by ELISA for *E. coli* lysates expressing: (**B**) wild‐type FUS; (**C**) arginine substituted to serine for all RGG/RG domains, RS‐FUS; (**D**) truncation of the LC domain, ΔLC; and serine substituted only in the (**E**) second, RS2‐FUS, or (**F**) third, RS3‐FUS, RGG/RG domains. Biological replicates represent independent *E. coli* growth and inductions. Solid lines show data averaged from 2 biological replicates for RS3‐FUS and 3 biological replicates for all other proteins expressed. Dashed lines indicate standard error about the mean (SEM).

BL21(DE3) cells were transformed with an inducible expression plasmid for FUS fused with N‐terminal 6xHis and maltose‐binding protein (MBP‐FUS). After MBP‐FUS expression was induced, transformed *E. coli* were grown overnight at 17°C as previously described for optimal and stable expression. Cells collected were divided and half crosslinked with formaldehyde (*see Methods*) or not crosslinked [14]. Cell lysates were sonicated and incubated with benzonase, a nuclease for DNA and RNA, so that small protein complexes could not appear larger by binding nucleic acids. After SEC, the size of particles in fractions were measured using dynamic light scattering (Figure 1A). Fractions were assigned to three groups according to size and elution volume (EV). Small fractions ranged from 18 to 26 mL EV and contained particles < 50 nm in diameter. Large fractions eluted in early volumes, 8 to 13 mL EV, with particles >150 nm in diameter. Medium fractions ranged from 13 to 18 mL EV containing particles of 50–150 nm in diameter.

MBP‐FUS was measured in fractions using ELISA. Its elution profile matched that found previously for endogenous FUS in human cells (Figure 1B) [23]. MBP‐FUS eluted mostly in small particles for uncrosslinked lysates and large particles in crosslinked lysates. We determined whether self‐assembly was diminished in *E. coli* by truncations or amino acid substitutions targeted at the LC or RGG/RG domains, as previous studies have shown [36, 42]. MBP‐FUS with all arginine residues replaced by serine (RS‐FUS) eluted in small particles in the crosslinked samples (Figure 1C). A deletion of the LC domain (ΔLC) produced crosslinked assemblies that eluted with medium‐sized particles (Figure 1D) [23, 43]. A substitution of arginine residues in the second RGG/RG domain (RS2‐FUS) produced assemblies broadly distributed in size (Figure 1E). Finally, substitution in the third RGG/RG domain (RS3‐FUS) did not produce assemblies of large or medium size (Figure 1F) [35].

### 3.2 MBP‐FUS Assemblies Detected in *E. coli* by SEC‐MS

**FIGURE 2:**
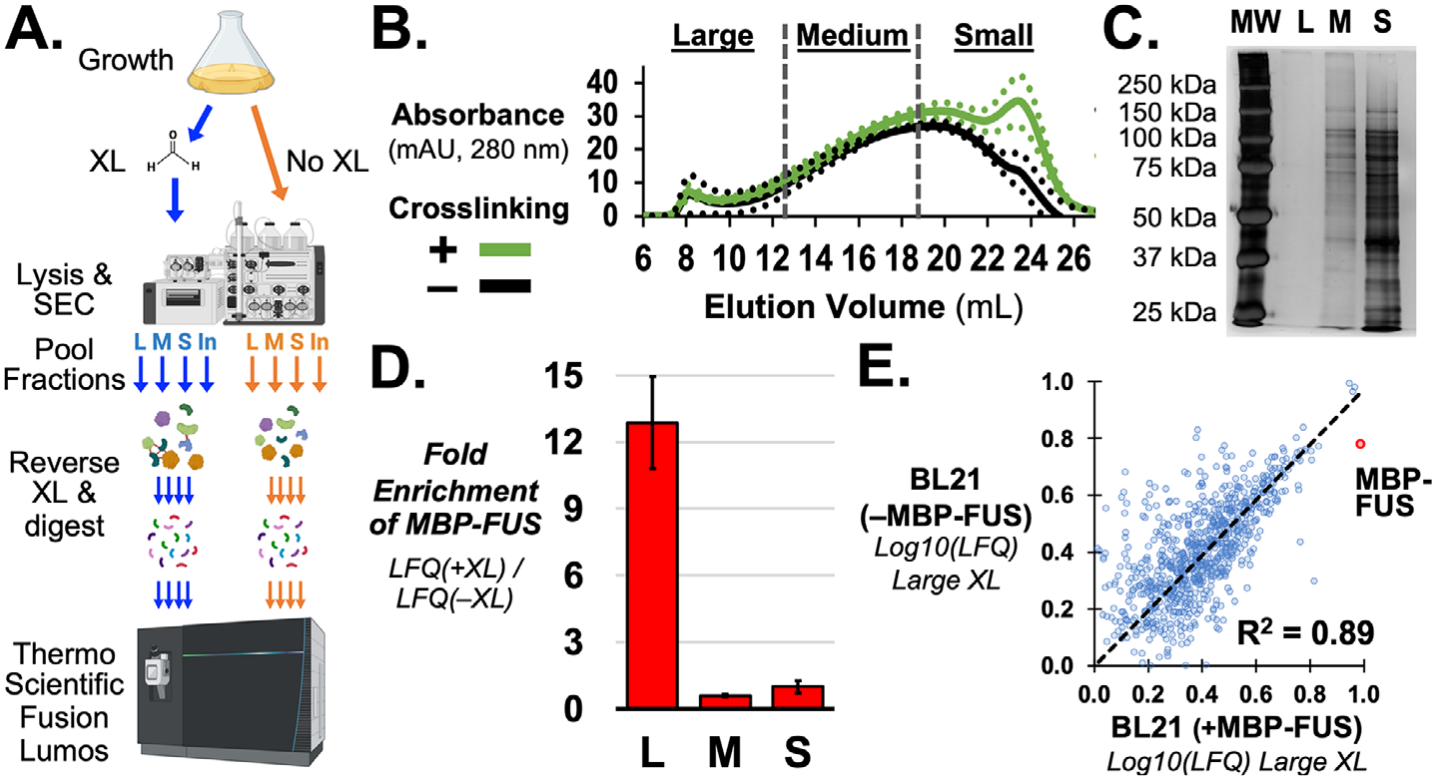
**SEC‐MS can detect crosslinked FUS particles formed in *Escherichia coli***. (**A**) Outline of experiment design and SEC‐MS protocol steps (*created with BioRender.com*). (**B**) Absorption measured during SEC for crosslinked and uncrosslinked *E. coli* lysates. Dashed lines show standard error about the mean (SEM). (**C**) Silver stained SDS‐PAGE gel of protein precipitated and resuspended from fraction pools: large, “L”; medium, “M”; and, small, “S”. MW indicates molecular weight marker. (**D**) Fold enrichment of MBP‐FUS in crosslinked lysates, LFQ(+XL), relative to no crosslink, LFQ(–XL). Error bars show standard error about the mean (SEM). (**E**) Scatter plot comparing crosslinked protein levels in large pools from *E. coli* lysates that contained and expressed the *MBP‐FUS* gene, +MBP‐FUS, or empty plasmid, –MBP‐FUS. The log_10_ of LFQ is normalized to the sample's max value and values are averaged from MBP‐FUS (N = 2) or empty plasmid (*N* = 3). The red point shows MBP‐FUS levels compared to the same endogenous malE protein.

For SEC‐MS, MBP‐FUS expression was induced as above and divided to samples with and without crosslinking (Figure 2A). After SEC, the large, medium, and small particle fractions were pooled then TCA precipitated. After reversal of formaldehyde crosslinks, proteins were digested in solution for analysis by label‐free quantitative proteomics. This process was repeated for *E. coli* transformed with plasmid not expressing MBP‐FUS (empty plasmid).

UV absorption (280 nm) during SEC did not find crosslinking to strongly affect total protein elution (Figure 2B) [23]. Analysis by SDS‐PAGE and silver‐staining indicated diverse protein populations in medium and small pools (Figure 2C, Figure S1A). Western blot analysis of FUS elution after reversal of crosslinking showed a similar elution as ELISA with some residual smearing of bands due to formaldehyde (Figure S1B). The proportion of peptides in the three pools only showed small differences: 7.9 ± 3.3% to 10.0 ± 4.8% for large uncrosslinked and crosslinked pools, 21.2 ± 6.4% to 26.7 ± 6.4% in medium pools, and 70.9 ± 8.4% to 63.2 ± 8.7% in small pools (*N* = 5, Table 1). Minimization of DNA in samples by sonication and benzonase treatment was confirmed by agarose gel electrophoresis stained with ethidium bromide (Figure S2A).

**TABLE 1:** The percentage of total peptides for each pool of SEC fractions from samples with and without crosslinking.

	Avg(–XL)	SD(–XL)	Avg(+XL)	SD(+XL)
Large	7.9%	3.3%	10.0%	4.8%
Medium	21.2%	6.4%	26.7%	6.4%
Small	70.9%	8.4%	63.2%	8.7%

Proteomic analysis of pooled fractions identified 2094 *E. coli* proteins in input samples and 1838 proteins in the small pool (Table 2, Table S1). Large and medium pools contained 1145 and 1764 proteins, respectively. Enrichment by crosslinking was taken to be the ratio of crosslinked raw intensities to uncrosslinked samples. Differential expression (DE) analysis assumes that relative LFQ changes are predominantly small and distributed equally about a geometric mean [44]. To correct DE analysis to reflect relative enrichment, LFQ was normalized about the first quartile or median of values below the geometric mean. The normalization had little effect on input and small pools, while negative enrichment measured relative to the geometric mean was reduced Figure S2B). Most proteins enriched >2‐fold were found in large (*N* = 754) and medium (*N* = 896) particle pools, of which 313 (large) and 854 (medium) were significant by *t*‐test (Table 2). Very few significant effects from crosslinking were detected in small pools (*N* = 95) or input samples (*N* = 9).

**TABLE 2:** Number of proteins detected and quantified in pooled fractions eluted from SEC.

	Large	Medium	Small	Input
Particle size(nm)	>150	150‐50	<50	NA
Proteins detected	1145	1764	1838	2094
>2‐fold enriched	754	896	116	14
>2‐fold enriched (p<0.05)	313	854	95	9

LC‐MS/MS showed 12.9‐fold enrichment of MBP‐FUS in crosslinked large particles (*N* = 3, Figure 2D, Figure S3A). MBP expressed in (+MBP‐FUS) cells was considerably more enriched in large particle than endogenous malE expressed in (–MBP‐FUS) cells (Figure 2E **)**. Comparison of MBP‐FUS transformed cells versus empty plasmid revealed a strong correlation in expression levels of endogenous *E. coli* proteins (*R* ^2^ = 0.89). Strong correlations were also measured for enrichment by crosslinking in large (*R* ^2^ = 0.70) and medium (*R* ^2^ = 0.68) fraction pools (Figure S3B).

### 3.3 Analysis of *E. coli* Proteins in Large Particles

**FIGURE 3:**
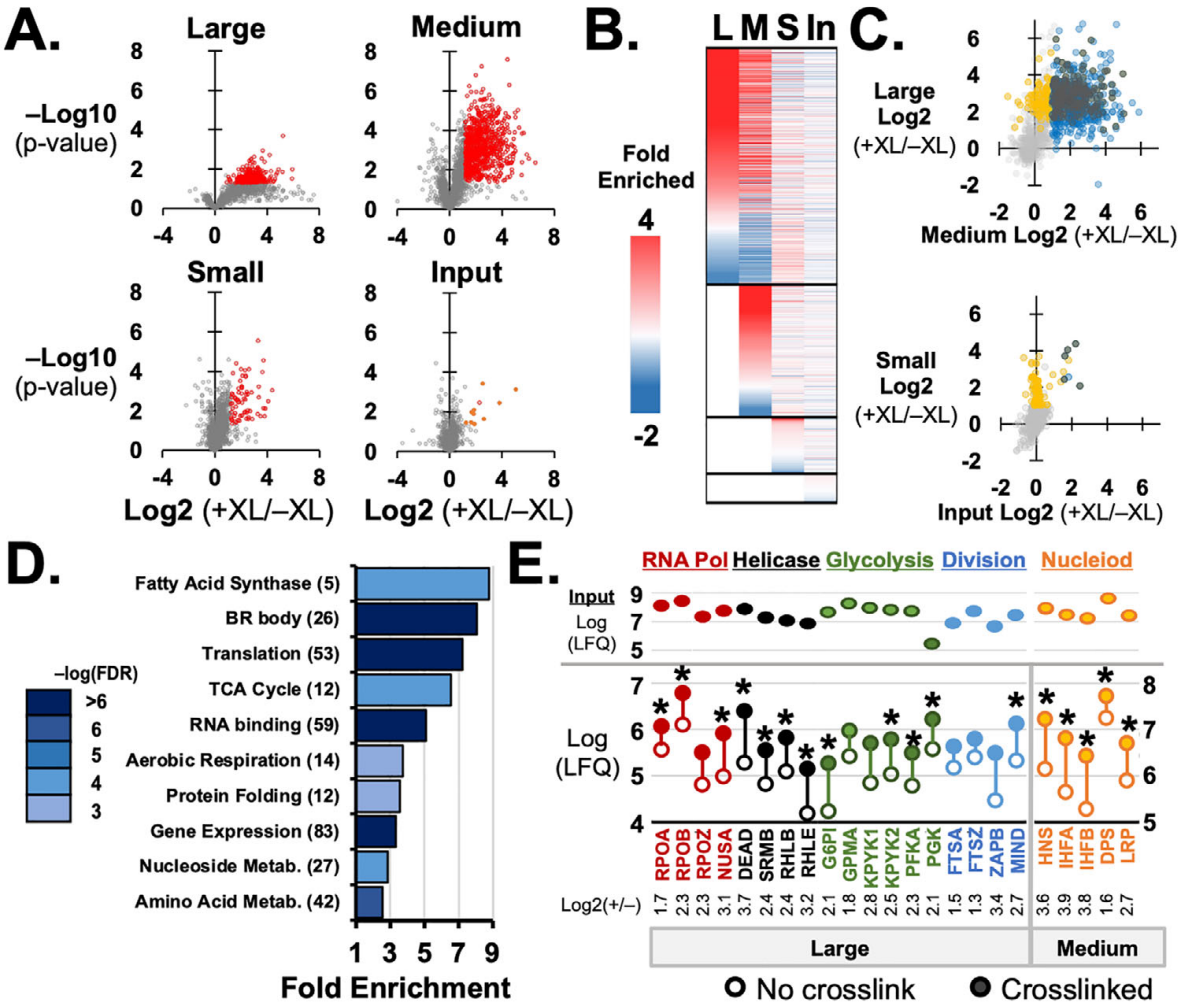
**Enrichment of *E. coli* proteins by crosslinking among large particles**. (**A**) Volcano plots for averaged fold enrichment by crosslinking and p‐value. Plots are shown for the large, medium, and small pooled SEC fractions, as well as input samples, for 3 independent *E. coli* growths. In red are proteins with >2‐fold enrichment and *p* ≤ 0.05 by Student's *t*‐test for equal variances. Number of proteins shown and significant are provided in Table 2. (**B**) Heat map showing fold enrichment and sorted by left most column. Lower sections show proteins that were not detected in large pools, or medium and large pools, or only detected in input samples. (**C**) Upper scatterplot shows fold‐enrichment >2‐fold (*p* ≤ 0.05) for large (yellow) versus medium (blue) particles and those significantly enriched in both (black). Lower scatterplot shows that for small (yellow) versus input (blue). (**D**) Bar plot shows fold enrichment for selected GO associations. False discovery rate (FDR) is indicated by the color gradient on the left. (**E**) Plot of raw LFQs for individual proteins measured with and without crosslinking for large particles (lower panel), input samples (upper panel), and grouped into class or cell processes of RNA Pol, helicases, glycolysis, and cell division machinery. LFQs for nucleoid proteins are from the medium‐sized pools and correspond to the right vertical axis. Asterisks (*) indicate *p*‐value ≤ 0.05, Student's *t*‐test for equal variances. Log2(+XL/‐XL) fold change values plotted along the bottom.

We next examined enriched *E. coli* proteins for those found in known prokaryotic subcellular structures [3, 13, 45]. The largest and most significant enrichment was found in the medium pools with 50–150 nm diameter particles and secondly in the large pool with particle diameters >150 nm (Figure 3A, Table S1). Very few large or significant effects were seen among ordinary‐sized protein complexes in the small pool. Proteins enriched in large pools were likely to also be found enriched in medium pools (Figure 3B) with a high correlation in enrichment (*R* ^2^ = 0.56, *p* << 0.001, Figure 3C). Combining large and medium pools, 216 proteins significantly enriched by >2‐fold. Of proteins significantly enriched in medium pools, 266 were enriched in large pools by >2‐fold but did not reach significance (*p* > 0.05).

We inquired whether enrichment by crosslinking could demonstrate predicted enrichment in gene ontology (GO) annotations using PANTHER or suggest novel interactions among large and medium particles. Among *E. coli* proteins enriched in the large pools, PANTHER found a wide range of cellular components and processes enriched (Figure 3D, Table S2). These included metabolic synthesis pathways for amino acids, nucleotides, and fatty acids. Also enriched were protein members of energy converting metabolic pathways, including TCA cycle and aerobic respiration [3, 46]. Large pools were enriched for RNA binding proteins and protein chaperones, consistent with the makeup of cell bodies in eukaryotic cells (Figure 3D). Combined enrichment of large and medium pools added significance to GO associations (Table S2). For example, the number of large and medium pool proteins associated with protein folding (*N* = 29) was more than double the large pool alone (*N* = 12). The combined pool provided more specific associations, such as protein chaperones (unfolded protein binding) and proline isomerases, rather than the generic annotation of protein folding (Table S2).

Among the most significant enriched annotations identified by PANTHER was gene expression (Figure 3D). Recent studies have also drawn attention to these prokaryote subcellular structures [47, 48]. An imaging‐based study found RNA Pol subunit RpoC and transcription factor nusA in biomolecular condensates in *E. coli*. Our study identified 5 out of 6 RNA Pol subunits and 70 out of 96 known RNA Pol interactors were significantly enriched in large and medium pools (Table 3, Figure S4A). BR body proteins were also enriched. For BR body protein RNase E, rne, 75 interactors were observed among small and medium particles and 43 of these were significantly enriched by crosslinking (Table 3, Figure S4B).

**TABLE 3:** Proteins identified by IntAct as protein complex interactors and enriched in large or medium particles. Previous work indicates components observed by an orthogonal method to be incorporated into a similar or larger complex as measured by SEC‐MS.

Complex	Query	IntAct	Seen^a^	(FE) >2^1*^	Proteins Enriched^1*^ (excluding ribosomal proteins)	Prev. Work	Ref.
RNA polymerase	RpoA	96	80	70	coaBC, cspA, cspC, dbhA, dpbB, dpo1, fur, g3p1, GreA, hfq, nfi, nusA, nusG, obg, odp1, odp2, pnp, pta, rf2, rho, rp54, RpoA, RpoB, RpoS, RpoZ, ssb, sspA, syd, top1, TpiS, tsaD, yacL, ybbO	RpoC, NusA	**[** 2, 13, 47, 48 **]**
BR body	rne	84	75	43	cra, crp, deaD, eftu1, eno, fabZ, hfq, minD, odo2, odp2, pgk, rhlB, rluB, rne, slyD, srmB, tkt2, yecA, yfiF	rne, rsaA	**[** 45, 46, 53 **]**
Degradosome	Rhle	36	31	28	clpP, cspA, cspC, dbhB, fer, frmA, glaR, if2, ipyr, nadE, nfsB, pdxH, Rhle, rluC, rpoB, sodM, ssb	RhlE	**[** 49, 50, 54 **]**
Z‐ring & divisome	FtsZ	96	63	35	acrR, add, btuR, ch60, ClpX, CRP, efg, eftu1, fepB, ftsA, ftsE, glyA, gnsA, htpG, hslU, htpG, ibpA, minC, minD, mpl, murD, odo2, ppk1, secA, thio, thio2, uspG, zapA, zapB, zapD	FtsZ	**[** 51, 55, 56 **]**
Nucleoid	h‐ns	101	70	54	astE, bluR, dtd, dut, flaV, gch1L, hgfr, hha, hns, mak, malQ, msyB, nagB, phsM, pyrH, secA, slyD, stpA, tig, uspA, yebC, yeeN (+23 proteins^c^)		**[** 2, 52, 57, 58 **]**

^a^ Large and medium particle sizes.

^b^ *p*<0.05 Student's *t*‐test, assume equal variances.

^c^ additional interactors in Table S2.

Manual curation with assistance of published literature extracted more proteins known to comprise large subcellular structures. In all cases inspected, significantly enriched proteins in large and medium size pools were also present and quantified in input samples (Figure 3E). Among RNA‐dependent DEAD‐box ATPases, Rhle has been observed in condensate structures and is a known constituent of the degradosome (Table 3) [49, 50]. Crosslinking enriched Rhle 9.2‐fold in large and 2.8‐fold in medium particles, with *p* = 0.015 and 0.0015 respectively (Student's *t*‐test for equal variances, Figure 3E). Other DEAD‐box proteins previously found in condensates and significantly enriched in large and medium particles in our findings were DeaD and SrmB [49]. Two additional DEAD‐box proteins that failed to form structures qualified as condensates by the same previous report were enriched in either large or medium particles. RhlB was significantly enriched in both large and medium particles, and DbpA was 2.9‐fold enriched in medium (*p* = 0.0025, Student's *t*‐test for equal variances) but not detected in large particles (Figure 3E).

Another approach to sort enriched proteins in large and medium particles was by interactomes. The protein FtsZ is a prominent constituent for two large *E. coli* structures involved in cell division, the divisome and Z‐ring [51]. We used FtsZ interactors deposited in the IntAct database and found 63 out of 96 were detected in large or medium particles (Table 3, Figure S4B). Of interactors enriched >2‐fold, 30 were significantly enriched. We repeated this approach for a key protein member of the *E. coli* nucleoid structure, h‐ns [52]. This highly abundant protein member to one of the largest structures observed in *E. coli* has 101 interactors, of which 70 were observed and 45 were significantly enriched between large and medium particles (Table 3, Figure S4B).

Finally, protein associations underrepresented in large and medium particles were determined by PANTHER. The most notable negatively enriched categories related to membrane bound proteins. In fact, the only underrepresented categories besides membrane transport proteins were those involved in viral and transposable elements, cell motility, adhesion, and unclassified (Table S2). These broad categories encompass many large and well‐studied protein structures, suggesting that their absence in our dataset may stem from a procedural or technical shortcoming.

To summarize, SEC‐MS with crosslinking can detect exogenous protein assemblies expressed in *E. coli*, quantitate enrichment for proteins comprising known cellular structures, provide evidence of expanded cell structure interactomes, and indicating protein structures, both novel and aligned with previous predictions. We conclude that SEC‐MS with crosslinking offers a useful approach to broadly survey constituents of protein structures in bacteria.

## 4 Discussion

In this study, SEC‐MS was assessed as an efficient and quantitative tool to distinguish candidate members of subcellular structures from protein complexes of ordinary size. Our approach analyzed enrichment due to formaldehyde crosslinking relative to noncrosslinked samples. We confirmed that the human FUS protein formed assemblies when expressed in *E. coli*, closely matching those formed endogenously in a human cell. The separation of FUS assemblies during SEC was observable by both ELISA and mass spectrometry. Through SEC‐MS, *E. coli* protein abundances showed hundreds of proteins significantly enriched by crosslinking in particles >50 nm in diameter. In this way, previously known subcellular structures could be confirmed. Additional enriched proteins within a functional category or interactome offer opportunities to predict novel protein assemblies contributing to *E. coli* biology [12, 13].

Several well characterized eukaryotic subcellular organelles can be extracted nearly intact from cell lysates. Examples include nucleoli [18, 59], stress granules [60], and p‐bodies [16]. Some prokaryotic organelles also have specific enrichment protocols, such as BR bodies [61]. The challenge in studying nonmembrane bound organelles is the weak interactions that make up their structure do not easily survive cell lysis. The method we demonstrate here circumvents this challenge by employing a chemical crosslinker, formaldehyde. Use of more specific chemical crosslinkers may elucidate the type of molecular interaction enriched through SEC. Nevertheless, formaldehyde is the most used chemical crosslinker, easily accessible to most labs, and a reagent in protocols standard across many disciplines of the biomedical and life sciences [20, 30, 34, 62].

Presently, there are a limited number of approaches to discover novel subcellular structures in cells. Historically, electron and fluorescence microscopy provided the evidence of cell structures not partitioned by lipid membranes. Innovations in super resolution microscopy have improved sensitivity and enabled inquiries into the types of molecular interactions involved. However, reliance on fluorescent tags, chemical labels, or immunofluorescence limits the range in target molecules specified by the experiment design. Our method measures total proteins enriched in large particles in an unbiased way. Although interactomes or localization would need be determined by subsequent experiments, SEC‐MS offers an inventory of likely constituents of subcellular structures and indicates how biological or genetic context might alter these.

The structural makeup of cell structures described as aggregates, condensates, or membrane‐less organelles necessitates that they be heterogeneous in their size and makeup. Resolution for protein SEC is typically described in terms of molecular weight for an idealized globular protein, such as 5–1500 kDa for a sec‐1000 column or 5–5000 kDa for the Superose 6 column [30, 33, 34]. For comparison, the bacterial ribosome is ∼20 nm in diameter and 2500 kDa, and the human RNA Pol II holoenzyme with Mediator complex is ∼25 nm on its longest axis and 4000 kDa [14, 23]. Our approach is to use the largest available SEC fractionation range, the CL2B, for coarse gel filtration of particles up to >300 nm in diameter. At these sizes, molecular weight is no longer a useful descriptor since the structures typically are not solely comprised of protein and mostly water by volume [9, 23]. Nevertheless, protein enrichment at this coarse scale resolution is important to understand both biology and disease. One example would be pathologic protein and nucleic acid bodies observed in amyotrophic lateral sclerosis (ALS), Huntington disease (HTT), or Type 2 diabetes [63]. The focus of this study is cell bodies in the prokaryote, *E. coli*, but more widely known bodies found in eukaryote cells include the nucleolus for ribosome assembly, Cajal bodies for rRNA modification, stress granules for mRNA regulation, and transcription condensates that organize RNA polymerases and splicing machinery [7, 10, 64]. The need to provide structure to organize on a gene RNA polymerase and spliceosome highlights that the condensates involved need be much larger than either protein complex, which is the reason that fine resolution SEC methods fall short [65].

This study provides the first proteome of formaldehyde crosslinking–assisted SEC–MS in a bacterial system. This data provides an opportunity to assess method optimization for future studies. First, the maximal protein concentration injected can increase SEC resolution, increase protein yield for MS analysis, but also clog filters. For this reason, 0.75 mg of protein was selected because minimal loss of FUS protein was observed by 0.45 µm filtration prior to SEC. However, two injections were needed for sufficient protein in the large no crosslink fractions for MS analysis. Although FUS protein remained in the soluble fractions during lysis and filtration, we noted in analysis of MS results that membrane proteins were underrepresented in fractions and input samples. We hypothesize this is due to solubility during sample preparation. Previous studies of membrane proteins by SEC‐MS suggests that optimizing lysis and detergents compatible with SEC may be an option to overcome this limitation [30, 33]. Last, the CL‐2b SEC column offers a cruder resolution than common SEC options. Although this study did not press the limits for MS analysis, the relative enrichment of proteins among the three pools and the resolution observed in the ELISA analysis suggests that future studies may find room for improvement. One last challenge faced is the lack of known size standards for cellular structures larger than a ribosome. However, this gap in knowledge is a compelling reason to continue studies of new approaches like that proposed here.

In conclusion, we have explored the potential for SEC‐MS as an unbiased method to inventory and quantify cellular proteins comprising large membrane‐less structures. We anticipate this method to be applicable for a broad range of species, cell type, and proteomes. Future directions might address this protocol's challenges with detecting membrane‐bound proteins, explore responses to a range of external conditions or treatments, determine cell type specificity in multicellular organisms, and compare disease and nondisease states in human cells. Further development in unbiased protocols is one approach to assess broad contexts in a cell's structure prior to designing a targeted study of a specific protein comprising one or more structures.

## Conflicts of Interest

The authors declare no conflicts of interest.

## Supporting information




**Supporting File 1**: pmic70105‐sup‐0001‐FiguresS1‐S4.pptx.

**Supporting File 2**: pmic70105‐sup‐0002‐TableS1.xlsx.

**Supporting File 3**: pmic70105‐sup‐0003‐TableS2.xlsx.

## Data Availability

The mass spectrometry proteomics data have been deposited to the ProteomeXchange Consortium via the PRIDE partner repository with the dataset identifier PXD054099 anhttps://doi.org/10.6019/PXD054099.
