# Supplementary material for: Identifying Subcellular Structure Components in Escherichia Coli by Crosslinking and SEC‐MS
Source: Proteomics. 2026 Jan 21;26(5):27–36. doi: 10.1002/pmic.70105 (PMC13106914; doi:10.1002/pmic.70105)
Supplement: Supplementary file 1 — Supporting File 1: pmic70105‐sup‐0001‐FiguresS1‐S4.pptx. [file PMIC-26--s003.pptx]

## Slide 1
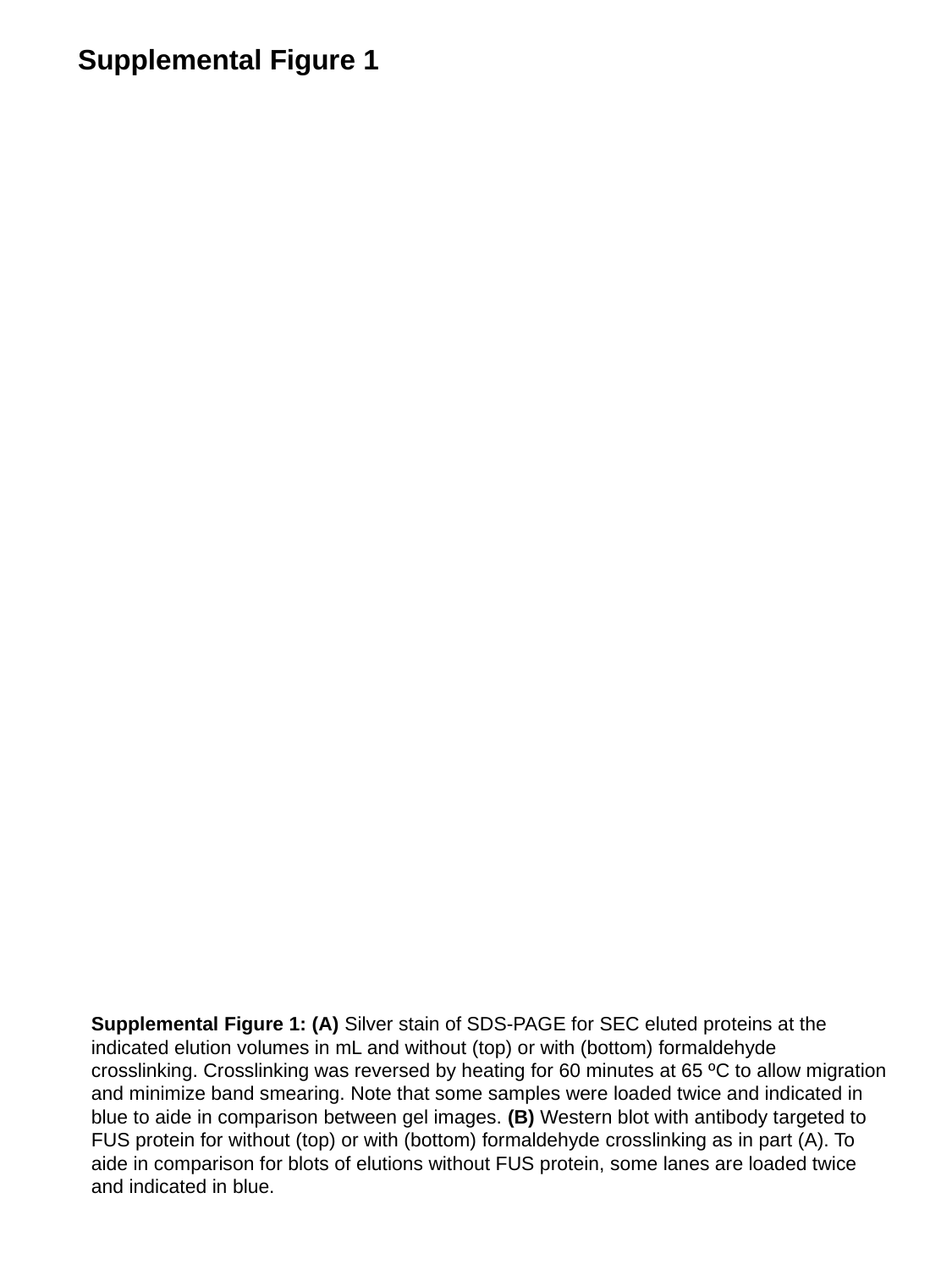

# Supplemental Figure 1
Supplemental Figure 1: (A) Silver stain of SDS-PAGE for SEC eluted proteins at the indicated elution volumes in mL and without (top) or with (bottom) formaldehyde crosslinking. Crosslinking was reversed by heating for 60 minutes at 65 ºC to allow migration and minimize band smearing. Note that some samples were loaded twice and indicated in blue to aide in comparison between gel images. (B) Western blot with antibody targeted to FUS protein for without (top) or with (bottom) formaldehyde crosslinking as in part (A). To aide in comparison for blots of elutions without FUS protein, some lanes are loaded twice and indicated in blue.

## Slide 2
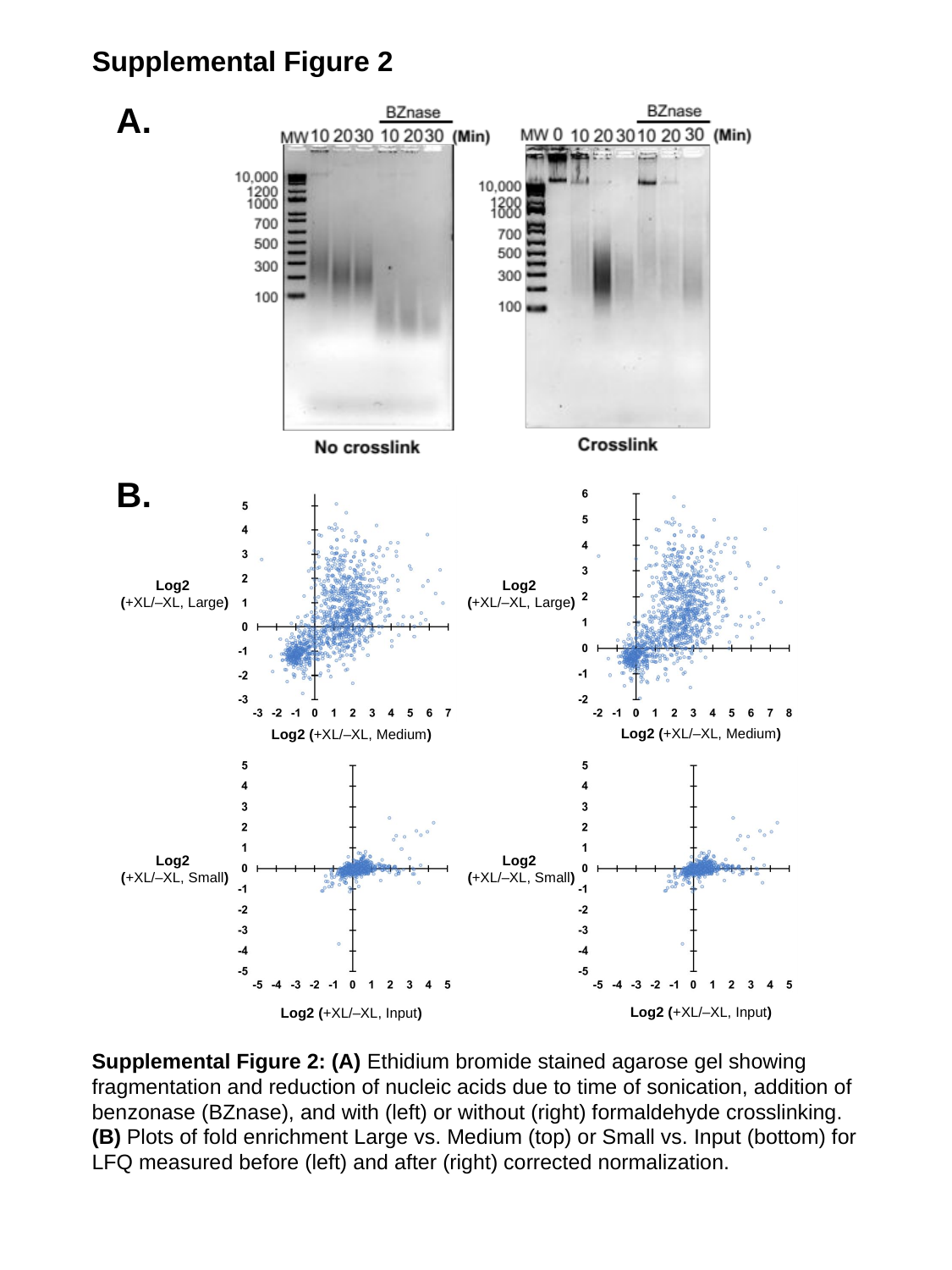

# Supplemental Figure 2
A.
B.
Log2
(+XL/–XL, Large)
Log2
(+XL/–XL, Large)
Log2 (+XL/–XL, Medium)
Log2 (+XL/–XL, Medium)
Log2
(+XL/–XL, Small)
Log2
(+XL/–XL, Small)
Log2 (+XL/–XL, Input)
Log2 (+XL/–XL, Input)
Supplemental Figure 2: (A) Ethidium bromide stained agarose gel showing fragmentation and reduction of nucleic acids due to time of sonication, addition of benzonase (BZnase), and with (left) or without (right) formaldehyde crosslinking. (B) Plots of fold enrichment Large vs. Medium (top) or Small vs. Input (bottom) for LFQ measured before (left) and after (right) corrected normalization.

## Slide 3
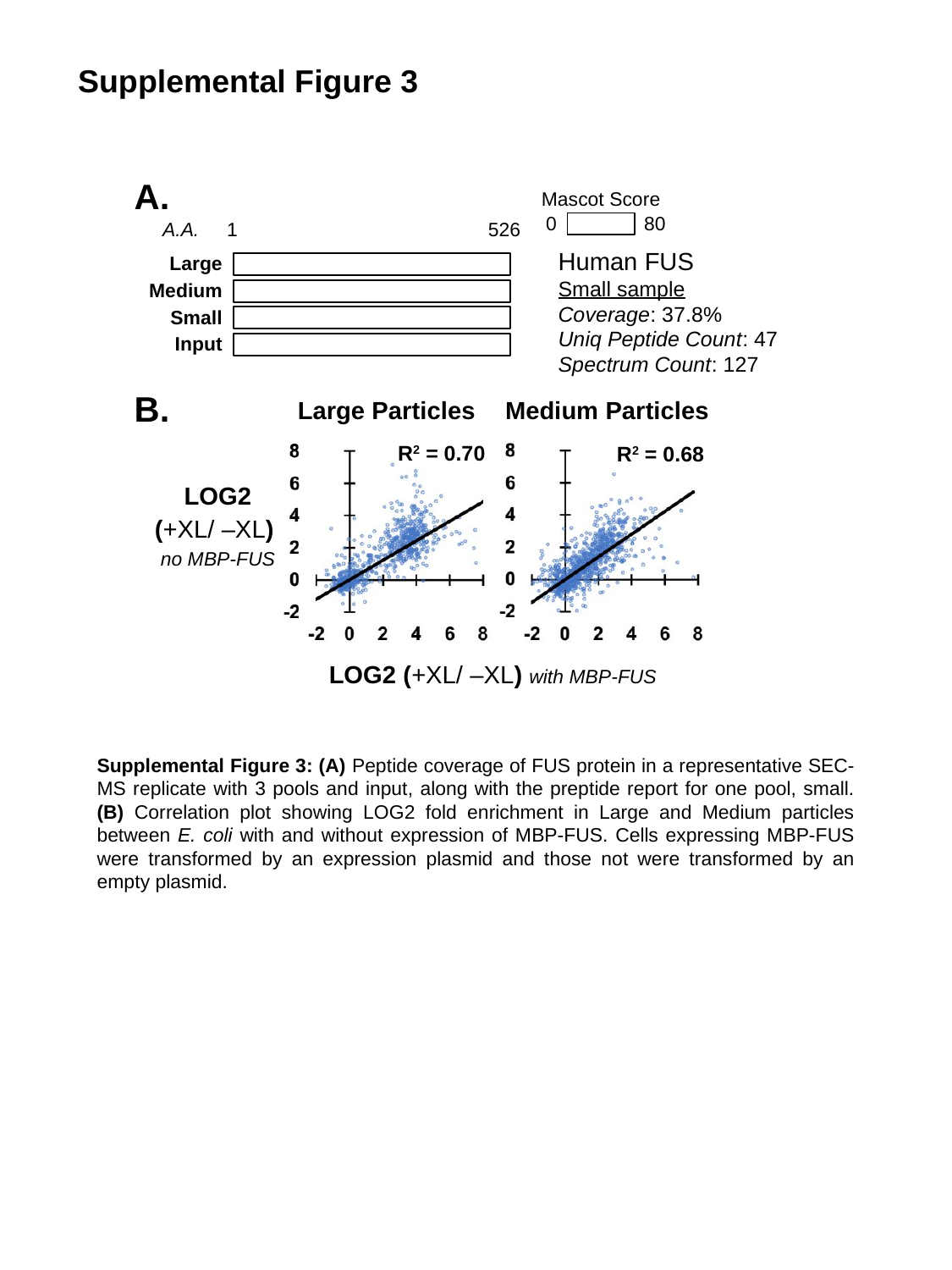

# Supplemental Figure 3
A.
Mascot Score
0
80
A.A. 1
526
Human FUS
Small sample
Coverage: 37.8%
Uniq Peptide Count: 47
Spectrum Count: 127
Large
Medium
Small
Input
B.
Medium Particles
Large Particles
LOG2
(+XL/ –XL)
no MBP-FUS
LOG2 (+XL/ –XL) with MBP-FUS
R2 = 0.70
R2 = 0.68
Supplemental Figure 3: (A) Peptide coverage of FUS protein in a representative SEC-MS replicate with 3 pools and input, along with the preptide report for one pool, small. (B) Correlation plot showing LOG2 fold enrichment in Large and Medium particles between E. coli with and without expression of MBP-FUS. Cells expressing MBP-FUS were transformed by an expression plasmid and those not were transformed by an empty plasmid.

## Slide 4
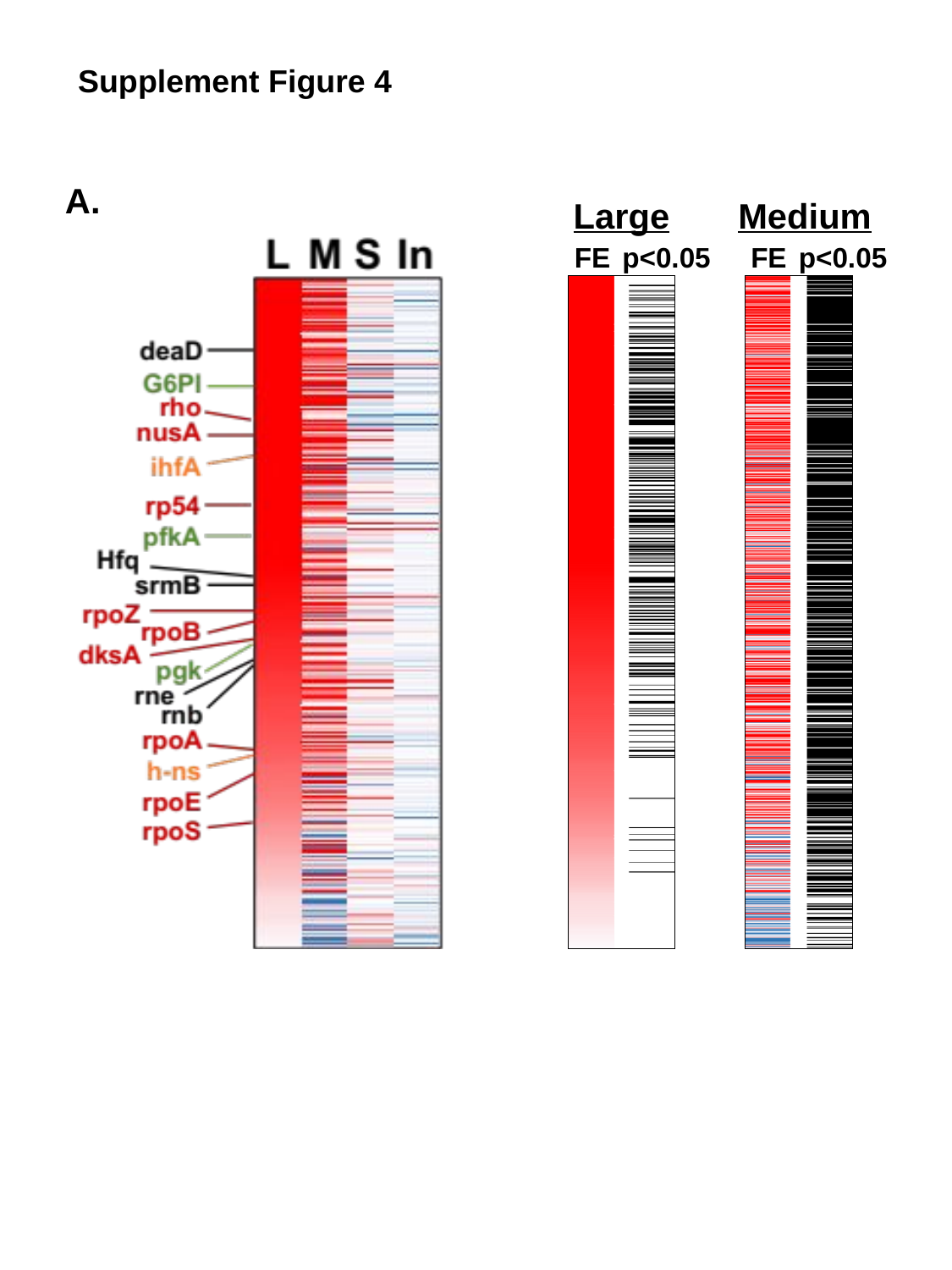

# Supplement Figure 4
A.
Large
Medium
FE p<0.05
FE p<0.05

## Slide 5
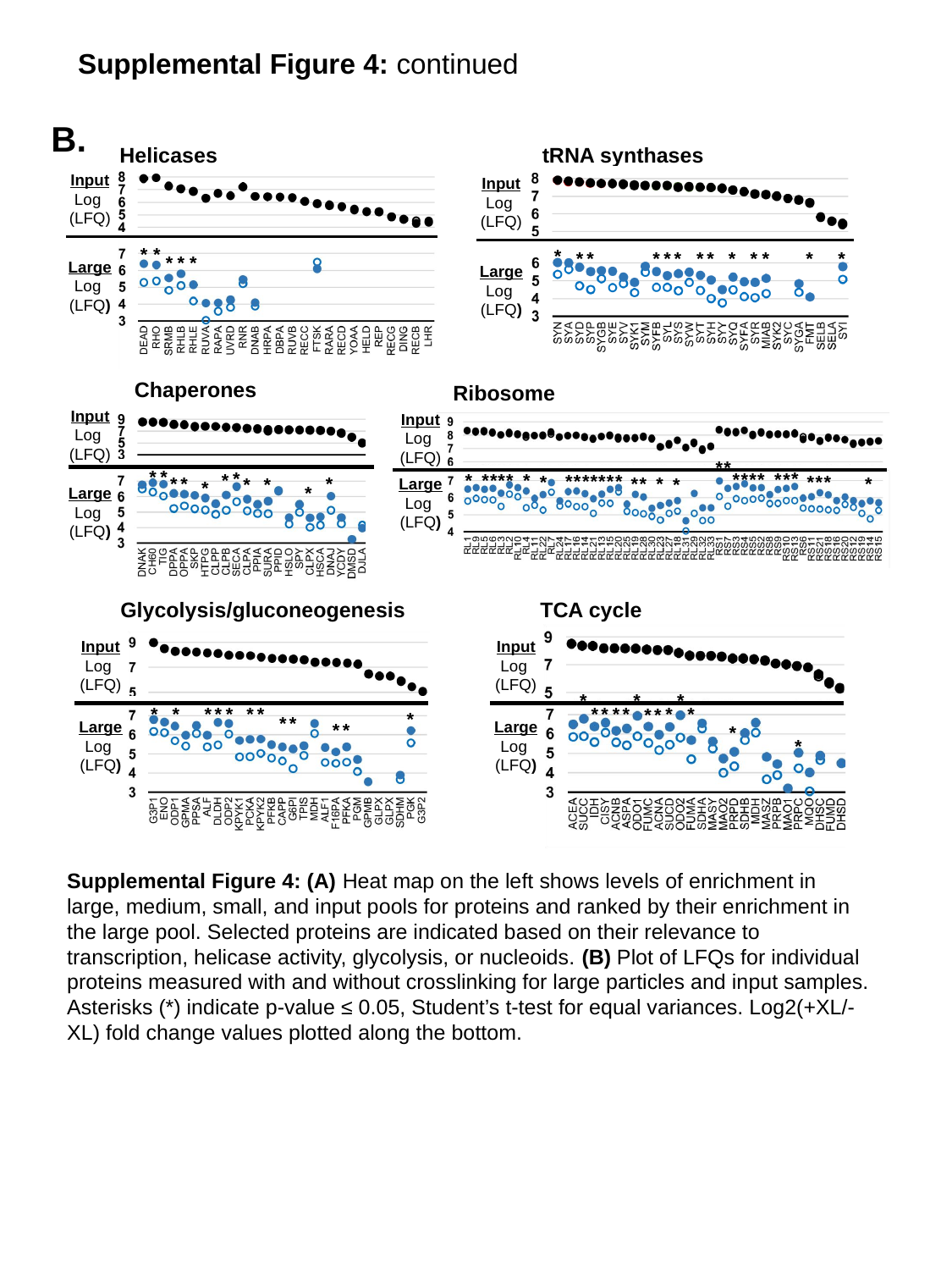

# Supplemental Figure 4: continued
B.
Helicases
Input
Log
(LFQ)
Large
Log
(LFQ)
*
*
*
*
*
tRNA synthases
Input
Log
(LFQ)
Large
Log
(LFQ)
*
*
*
*
*
*
*
*
*
*
*
*
*
Chaperones
Input
Log
(LFQ)
Large
Log
(LFQ)
*
*
*
*
*
*
*
*
*
*
*
Ribosome
Input
Log
(LFQ)
Large
Log
(LFQ)
*
*
*
*
*
*
*
*
*
*
*
*
*
*
*
*
*
*
*
*
*
*
*
*
*
*
*
*
*
*
*
Glycolysis/gluconeogenesis
Input
Log
(LFQ)
Large
Log
(LFQ)
*
*
*
*
*
*
*
*
*
*
*
*
TCA cycle
Input
Log
(LFQ)
Large
Log
(LFQ)
*
*
*
*
*
*
*
*
*
*
*
*
*
Supplemental Figure 4: (A) Heat map on the left shows levels of enrichment in large, medium, small, and input pools for proteins and ranked by their enrichment in the large pool. Selected proteins are indicated based on their relevance to transcription, helicase activity, glycolysis, or nucleoids. (B) Plot of LFQs for individual proteins measured with and without crosslinking for large particles and input samples. Asterisks (*) indicate p-value ≤ 0.05, Student’s t-test for equal variances. Log2(+XL/-XL) fold change values plotted along the bottom.
